# Are the presence of MODIC changes on MRI scans related to “improvement” in low back pain patients treated with lumbar facet joint injections?

**DOI:** 10.1186/s12891-015-0688-x

**Published:** 2015-09-04

**Authors:** Matilde Bianchi, Cynthia K. Peterson, Christian W. A. Pfirrmann, Juerg Hodler, Jennifer Bolton

**Affiliations:** Private Practice & Chiropractic Medicine Department, Orthopaedic University Hospital Balgrist, University of Zürich, Forchstrasse 340, 8008 Zürich, Switzerland; Departments of Radiology and Chiropractic Medicine, Orthopaedic University Hospital Balgrist, University of Zürich, Forchstrasse 340, 8008 Zürich, Switzerland; Department of Radiology, Orthopaedic University Hospital of Balgrist, University of Zürich, Forchstrasse 340, 8008 Zürich, Switzerland; Department of Radiology, University Hospital, University of Zürich, Rämistrasse 100, 8091 Zürich, Switzerland; Research and Continuing Professional Development, Anglo-European College of Chiropractic, 13-15 Parkwood Road, Bournemouth, BH5 2DF UK

## Abstract

**Background:**

Modic changes (MC) have been linked with low back pain (LBP) and worse outcomes from some treatments. No studies have investigated the impact that MCs may have on patient outcomes from lumbar facet injections. Therefore, the purpose of this study is to investigate whether the presence of Modic changes is related to ‘improvement’ in patients undergoing imaging-guided lumbar facet injection therapy.

**Methods:**

Outcomes from 226 patients with MRI scans within 3 months of their imaging-guided lumbar facet injections were investigated to determine whether MCs are related to ‘improvement’ post injection. At 1 day, 1 week and 1 month post injection the Patients Global Impression of Change scale answers were collected by postal questionnaire. This was the primary outcome measure. The numerical rating scale for pain data was collected prior to treatment and at the same post injection time points. The MRI scans were independently evaluated by two examiners for the presence/absence of Modic changes and the type of Modic change if present. Kappa statistics were used for reliability of diagnosis analysis. Chi-squared test and logistic regression analysis tested MCs with ‘improvement’.

**Results:**

Intra- and inter-examiner reliability for the diagnosis of MCs was Kappa = 0.77 and 0.74. Intra- and inter-examiner reliability for categorizing MCs was K = 0.77 and K = 0.78.

At 1 month post injection 45.2 % of patients without MCs reported clinically relevant ‘improvement’ compared to 34.2 % of patients with MC I and 32.1 % of patients with MC II. However, this did not reach statistical significance. Logistic regression found that Modic changes were not predictive of ‘improvement’.

**Conclusions:**

There was a tendency for patients without MCs to have better outcomes but this did not reach statistical significance. The reliability of diagnosing MCs was substantial.

## Background

Low back pain (LBP) is a common, disabling and costly condition affecting the adult population. Currently 85 % of patients seeking care for LBP are classified as having non-specific LBP [1]. To provide better patient care, it has become important to try to identify specific subgroups within this heterogeneous LBP population which may require different or specific types of treatments.

Recently, Modic changes (MCs) have been one of the new topics of interest investigated in the current spine literature because of their suggested importance as a specific cause of LBP [2, 3]. Modic changes are recognized on magnetic resonance imaging (MRI) scans as alterations in signal intensity of the bone marrow adjacent to a degenerated disc. There are three types of Modic changes namely signs of bone marrow edema (Modic type I), fat (Modic type II) and osseous sclerosis (Modic type III) [4–7]. Modic type I (bone marrow edema) has specifically been linked to LBP [2, 3, 7]. If Modic changes are a specific cause of LBP it now becomes important to assess their influence, if any, on LBP treatments and outcomes from specific treatments. In 2011 Jensen and Leboeuf-Yde [4] performed a systematic critical literature review to investigate if there is evidence in the literature that the presence of MCs at baseline is associated with a favourable outcome depending on the treatment provided for LBP. The six studies found were too few, too heterogeneous and often lacking in adequate methodological rigour, to make a definitive conclusion as to if and how MCs are an indication for specific therapies for LBP. Since that time, there is one additional randomized controlled clinical trial (RCT) and two recent cohort studies [5–7] evaluating the link between MCs and clinical outcomes. The RCT compared rest therapy with exercise in patients with Modic changes and found no significant differences in outcomes between these two groups. However, the treatments were not compared with patients who did not have Modic changes. The cohort studies, using fairly large sample sizes, showed that the presence of MC type I changes, at least at baseline, was associated with a poor outcome. However, specific treatments for the LBP were not evaluated. Thus, investigation of the role that Modic changes may have on treatment outcomes in LBP patients remains in its infancy.

If Modic changes are a specific cause of LBP, it is hypothesized that patients with MC type I, the inflammatory stage of the disc degeneration process, may have less favourable outcomes from imaging-guided facet joint injections as these Modic changes may be an additional cause for their LBP. In the literature there are currently no studies clearly establishing factors linked with positive or negative outcomes in the intervention of lumbar facet joint steroid injections. Therefore the aim of this study is to try to further the research on the importance of MCs in a group of non-specific LBP patients treated with therapeutic imaging-guided lumbar facet joint injections.

The specific objectives for the study were: a) to determine the reliability of detecting MCs on magnetic resonance (MRI) scans in patients receiving imaging-guided lumbar facet injections; b) to determine whether there is a difference in outcome between LBP patients with and without MCs treated with therapeutic imaging-guided lumbar facet injections.

## Methods

### Ethics approval

Ethics approval was obtained for all imaging-guided injections and collection of patient follow-up self-reported data from the Orthopaedic University Hospital Balgrist and the Canton of Zürich ethics committees (EK 12/2009). Informed consent was obtained from every patient.

### Patients

Over 400 patients received imaging-guided lumbar facet injections from June 2009 up to April 2013 at this Hospital, and returned outcomes-based postal questionnaires. These lumbar facet injection patients are part of the large, ongoing, imaging-guided therapeutic injections outcomes database started at this hospital in 2009 as part of the quality assurance procedures. These questionnaires included the patient’s global impression of change (PGIC) scale, as well as the 0 to 10 Numerical Rating Scale (NRS) for pain intensity, where 0 = no pain and 10 = the worst pain imaginable. As this is a specialized university orthopedic hospital, the majority of patients are chronic.

Of these, a total of 226 patients met the inclusion and exclusion criteria for entry into the study. All included patients had lumbar spine MRI scans performed at this Hospital within 3 months of the imaging-guided facet injections. No distinction was made as to whether the MRI scans were performed 3 months before or after the intervention. The natural course of MCs is a long one, and thus three months was not considered long enough to influence the nature of the MC.

**Fig. 1:**
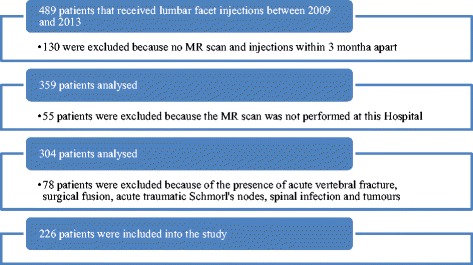
Flow chart of inclusion process

Exclusion criteria consisted of recent acute vertebral fractures, surgical fusions, acute traumatic Schmorl’s nodes, spinal infection or tumours. Patients for whom the facet injection procedure was a contraindication, such a pregnancy and anticoagulant therapy, were also excluded from the study. A flow-chart of patient exclusions is given in Fig. 1. Pain medication intake was not an exclusion criterion.

### Lumbar facet injection treatment

The interventions were done using fluoroscopy-guided therapeutic lumbar facet joint injections performed by musculoskeletal radiologists at this hospital. Under sterile conditions (3x disinfection, sterile gloves, mask, sterile covering), fluoroscopy-guided puncture of the relevant facet joint/s were performed. Documentation of the needle position was done with contrast medium and slice imaging followed by injection of 40 mg Kenacort (Triamcinoloni acetonium; Dermapharm AG, Huenenberg AG, Switzerland) and 1 ml Ropivacaine (Naropin; Astra-Zeneca, Södertälje, Sweden). The radiologists performing these injections report that it is almost always the most degenerated facet joint/s that are targeted for treatment based on the referral requests from the orthopedic surgeons and rheumatologists.

### MRI procedure

All MRI scans were performed at this Hospital where there are three MR scanners available (Siemens Magnetom Avanto 1.5 T, Espree 1.5 T or Verio 1.5 T). Sagittal T1 weighted, sagittal T2 weighted and transverse T2 weighted images were acquired. Protocol for the 1.5 T Avanto MR scanner: Sagittal T2 weighted (TR/TE 3740 ms/118 ms, field of view 300x300cm, slice thickness 4 mm, matrix 512x256 pixels), sagittal T1 weighted images (TR/TE 500 ms/11 ms, field of view 300x300cm, slice thickness 4 mm, matrix 512x256 pixels) and transverse T2 weighted sequences (TR/TE 3700 ms/115 ms, field of view 220x220cm, slice thickness 4 mm, matrix 512x256 pixels).

### Patient outcomes

Patient’s variables included age (measured in years) and sex (female vs. male). Data on the chronicity and the reasons for low back pain were not available but it is known that most patients referred to this specialized orthopedic university hospital are chronic patients.

Overall improvement was evaluated through the use of the PGIC scale taken at 1 day, 1 week and 1 month after injection. The PGIC is a 7-point categorical scale including responses from “much better” to “much worse”. For the purpose of the present study a priori definition of clinical importance consisted of only the scores of “much better” and “better” on the PGIC scales. These responses were considered clinically relevant “improvement” and this was the primary outcome measure. “Slightly better” was not considered as improvement. “Worsening” included the scores of “slightly worse”, “worse” and “much worse”. Based on this priori definition of clinical importance, the PGIC results were analyzed as a dichotomous categorical variable, 1 or 2 = yes (improved) and 3–7 = no (not improved). This is consistent with the use of this scale in several other studies [8–11]. The PGIC scale as the indicator of clinically relevant ‘improvement’ has been validated against other longer outcome measures [12]. The primary outcome was ‘improvement’ at 1 month but it was considered important to also evaluate outcomes at 1 day and 1 week as the infiltration contained both a short acting anaesthetic and the longer acting corticosteroid.

The NRS questionnaire was completed by each patient immediately prior to the injection procedure (baseline) and again at 15–20 min after injection. It was also completed at 1 day, 1 week and 1 month post injection. The NRS for pain is a standard instrument (numeric scale) which can be interpreted differently from patient to patient. To compensate for this variability the percent change in pain was calculated.

Unlike the NRS, the PGIC is specifically linked to the conceptual framework of overall improvement including disability levels. A close association between changes on the NRS and the PGIC has been demonstrated [12–14].

### Data collection

**Fig. 2:**
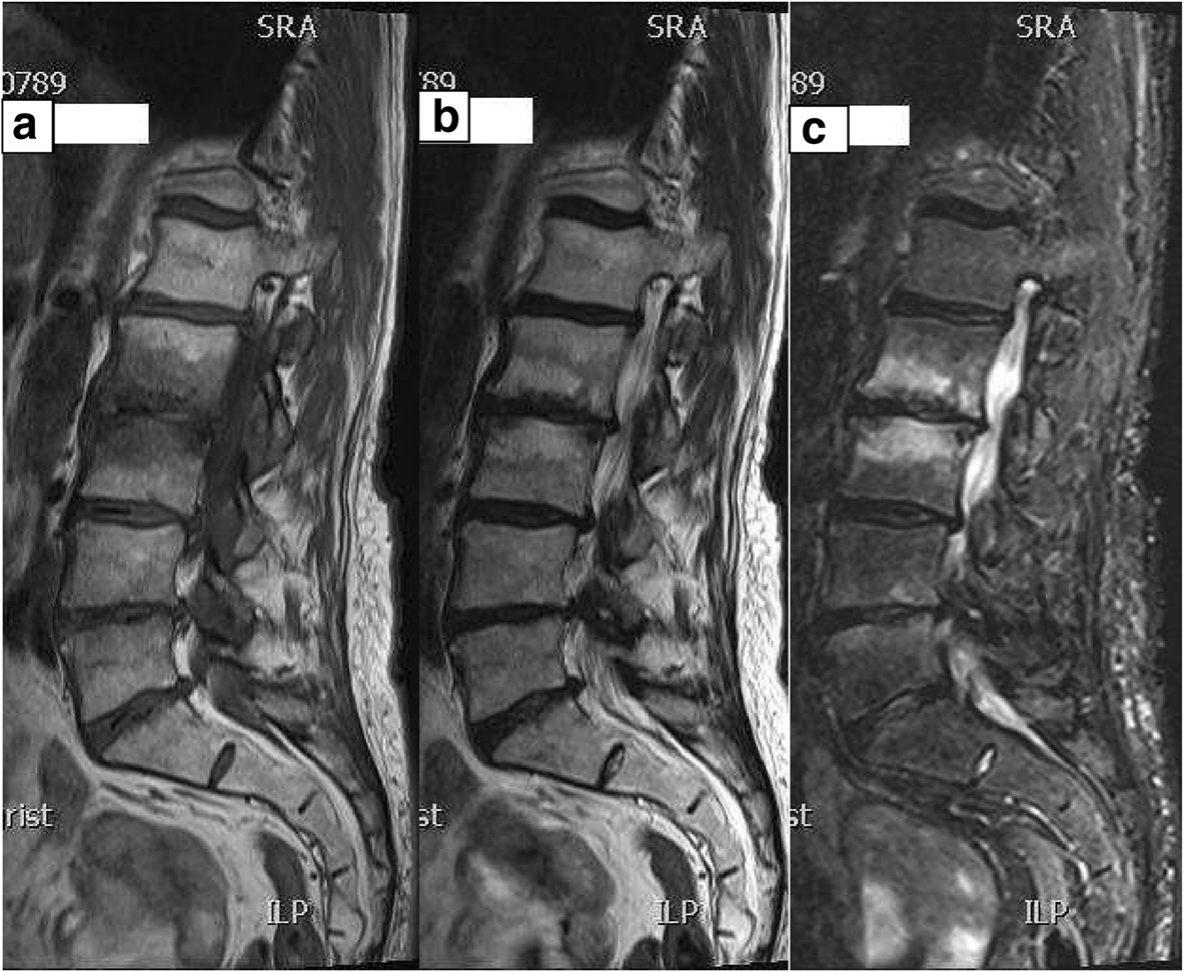
**a**, **b** and **c** Modic type I changes. T1, T2 weighted and fat suppressed sagittal MR images of a patient demonstrating low signal intensity adjacent to L2-3 disc on T1 weighted images (2a) and high signal intensity on both T2 weighted image (2b) and fat suppressed image (2c), consistent with MC type I (marrow edema) at L2 through 3

**Fig. 3:**
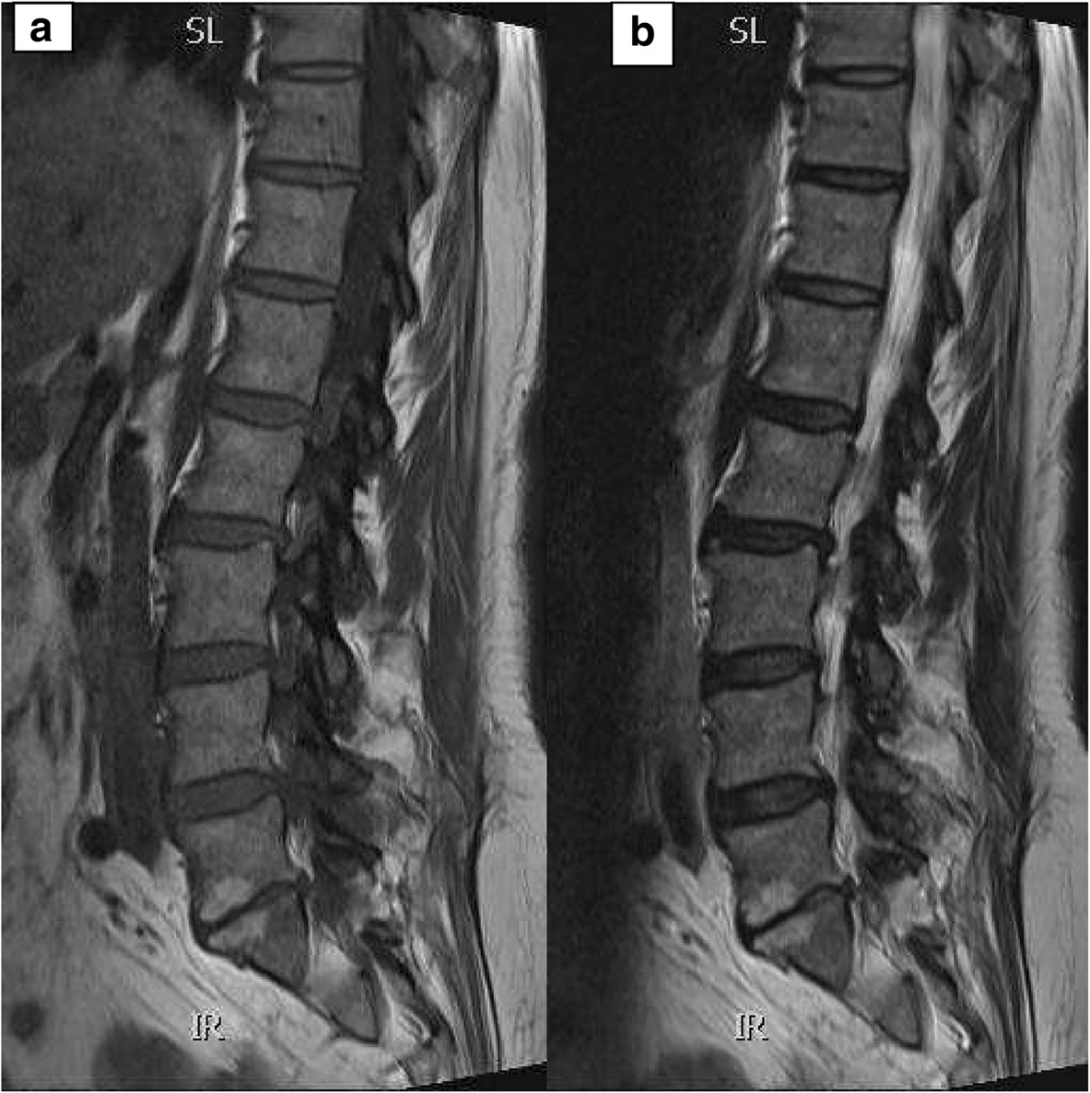
**a** and **b** Modic type II changes. T1 and T2 weighted sagittal MR images of a patient demonstrating high-signal intensity adjacent to the L5-S1 disc confirms MC type II marrow replacement. The L5-SI disc space is also narrowed

Data collection consisted of the evaluation of sagittal and axial T1-weighted sequences, T2-weighted sequences and fat suppressed images - when present - to determine:

- presence or absence of Modic changes and
- the type of Modic changes - type I (Fig. 2) and II (Fig. 3) only – if present. In cases showing MRI findings of both type I and II, the case was categorized as Modic type I.

The presence of Modic changes was reported as MCs vs. no MCs (YES/NO) and MC type I vs. MC type II (I/II). MC type III was not taken into consideration given its rarity.

The collection of MRI information was based on visual impression and was performed blinded to the patient outcomes by the first examiner (examiner 1). The first one hundred MRI examinations were re-evaluated by a second examiner (examiner 2) for inter-examiner reliability of detecting and categorizing MCs. This examiner was also blinded to the patient outcomes as well as to the MRI findings of examiner 1. Moreover the same one hundred of the MRI scans were re-evaluated more than 1 month after the initial MRI analysis for intra-examiner reliability by examiner 1. Finally, imaging characteristics were agreed by consensus in the few cases with disagreement.

### Statistical analysis

The SPSS version 17.0 program was used for data analysis and the statistical evaluation.

Descriptive statistics of age and gender were calculated.

Cohen’s Kappa statistic [15] was used to assess the intra- and inter-examiner reliability of the identification and categorization of MCs on MRI.

The Chi-squared test was used to explore the relationship between the presence/absence of MCs and type of MCs and “improvement” at 1 day, 1 week and 1 month. A *p* value < 0.05 was considered to be statistically significant. The presence and type of MCs were also compared for “worsening” using the Chi-squared test at 1 day, 1 week and 1 month.

For the purpose of the study the differences in pain as percent change in pain severity was calculated. The unpaired *t*-test was then used to compare the differences in change in pain intensity between patients with MC type I and patients without MCs, between patients with MC type I and patients with MC type II, and between patients with presence of MCs (type I and type II) and patients without MCs. Again a *p* value < 0.05 was considered to be statistically significant.

A logistic regression analysis was used to fit a predictive model given the variables of the study (independent variable: presence/absence of MCs; outcome variables: PGIC improvement yes/no). These results were presented as unadjusted and adjusted odds ratio (OR) with confidence intervals (CI). All values were adjusted for potential confounders (age and gender).

## Results

Between the dates of June 2009 and April 2013, 489 patients received imaging-guided lumbar facet joint infiltrations. Of these, 226 patients met the inclusion criteria for the study and 61.1 % were female patients. The mean age of all patients included in the study was 61.6 (SD ± 13.33) years with the youngest patient being 23 years old and the oldest patient being 88 years old. Modic changes were observed in 141 of the 226 patients examined (62.4 %). Of these, 83 were Modic change type I (36.72 %), 58 were Modic change type II (25.66 %) and 85 (37.61 %) presented with no Modic changes. There was a statistically significant difference in the ages when comparing patients with and without Modic changes. The mean age for patients without Modic changes was 56.6 (SD = 14.01) years, for Modic type I patients it was 64.8 (SD = 12.52) years (*p* = 0.001) and for Modic type II patients the mean age was 64.2 (SD = 14.01) years (*p* = 0.01). When using the Chi-squared test to compare gender with the various Modic categories there was no significant difference in the gender ratio between the categories.

There were no statistically significant differences in the baseline NRS scores when comparing patients with Modic I, Modic II, and no Modic changes. Modic I patients had a mean NRS score of 6.6 (SD = 2.37), Modic II patients reported a mean score of 6.9 (SD = 2.06) and patients without Modic changes had a mean score of 6.6 (SD = 2.19).

### Intra- and inter-examiner reliability

One hundred MRI scans were independently assessed by both examiners. The intra-examiner reliability for the presence/absence of MCs was K = 0.77 and for MC I vs. MC II K = 0.77 (*p* < 0.001). Inter-examiner reliability was K = 0.74 for presence/absence of MCs and K = 0.78 for MC I vs. MC II (*p* < 0.001). These results demonstrate substantial agreement for both intra- and inter- examiner reliability of detecting and categorizing MCs.

### Difference in outcome

The proportions (frequencies) of patients reporting “improvement” and “worsening” for the absence of MCs, MC type I only, and MC type II only and presence of MCs (type I and II) are shown in Table 1. The proportion of patients ‘unchanged’ (PGIC responses of ‘slightly better’ or ‘unchanged’) is not reported. A higher percentage of patients without Modic changes reported clinically relevant ‘improvement’ at 1 month post injection but this did not reach statistical significance.

**Table 1:** Difference in outcome

	No Modic changes (*N* = 85) %	Modic I (*N* = 83) %	Modic II (*N* = 58) %	Modic positive (Modic I and II) (*N* = 141) %	*P* value
Improved 1 Day	33.7 %	39.8 %	32.8 %	36.9 %	0.64
Improved 1 Week	41.5 %	40.2 %	37.5 %	39.1 %	0.37
Improved 1 Month	45.2 %	34.2 %	32.1 %	33.3 %	0.23
Worse 1 Day	10.8 %	9.6 %	5.1 %	7.7 %	0.44
Worse 1 Week	14.3 %	13.4 %	10.8 %	12.2 %	0.67
Worse 1 Month	17.0 %	14.0 %	16.1 %	14.8 %	0.66

Comparison of the proportion of patients with and without Modic changes reporting clinically relevant “improvement” or “worsening” at 1 day, 1 week and 1 month

There was a tendency for the subgroup of patients without MCs to maintain improvement obtained by the intervention longer in time as compared to patients with MC type I, MC type II and with the presence of MCs. Nevertheless, at one month after the intervention, the proportion of patients that reported a worse outcome increased in all subgroups.

### Prediction of outcome (improvement)

For the logistic regression analysis two different analyses were conducted: a) improvement (yes/no) in patients with MC type I were compared to patients without MCs, at 1 day, 1 week and 1 month and b) improvement (yes/no) in patients with MC type I were compared to patients with MC type II, at 1 day, 1 week and 1 month (Table 2).

**Table 2:** Association with outcome (improvement) Modic I vs. no Modic changes and Modic I vs. Modic II

	Modic I vs. No Modic Changes		Modic I vs. Modic II	
	Unadjusted OR (95 % CI)	Adjusted OR (95 % CI)	Unadjusted OR (95 % CI)	Adjusted OR (95 % CI)
1 Day	1.30 (0.69–2.44)	0.94 (0.48–1.86)	0.74 (0.37–1.49)	0.79 (0.37–1.57)
1 Week	0.82 (0.44–1.51)	0.69 (0.35–1.33)	0.89 (0.44–1.79)	0.94 (0.46–1.90)
1 Month	0.73 (0.39–1.39)	0.68 (0.35–1.32)	0.91 (0.44–1.90)	0.90 (0.43–1.89)

Unadjusted and adjusted (age and gender) odds ratios (OR) with 95 % confident interval (CI) for the association with improvement between Modic change type I and no Modic changes as well as Modic I compared to Modic II at the various time points

In both analyses the PGIC’ results obtained when controlling for age and gender for MC type I, at the three data collection time points, did not demonstrate a significant difference after lumbar facet injection therapy as compared to patients without MCs and as compared to patients with MC type II. However, a lower proportion of patient with MC type I reported “improvement”, but this did not reach statistical significance (wide confident intervals) when comparing these patients versus patients without MCs and versus patients with MC type II.

Evaluation for differences in percent change in pain severity (NRS scores) in the group with MC type I only vs. the no MCs group, the group with MC type I only vs. the group with MC type II only, and in the group with the presence of MCs of either type vs. the group with the absence of MCs are shown in Table 3. At one month, patients without MCs demonstrated a higher mean NRS change score than patients with the presence of MCs (11 % higher) and with MC type I only (14 % higher), but this did not reach statistical significance (*p* = 0.07).

**Table 3:** Differences in percent change in pain severity (NRS scores) Modic type I vs. no Modic changes, Modic type I vs. Modic type II and Modic changes present (both types I and II) and Modic changes absent

		Number	Mean	SD
% NRS Change 1 day	Modic I	81	35.46	39.35
	No Modic	85	34.90	35.29
% NRS Change 1 Week	Modic I	79	32.63	53.58
	No Modic	84	43.93	20.53
% NRS Change 1 Month	Modic I	77	22.99	56.50
	No Modic	81	36.97	36.89
% NRS Change 1 Day	Modic I	81	35.46	39.35
	Modic II	57	39.51	37.80
% NRS Change 1 Week	Modic I	79	32.63	53.58
	Modic II	55	45.19	39.68
% NRS Change 1 Month	Modic I	77	22.99	56.50
	Modic II	54	30.52	42.56
% NRS Change 1 Day	Modic I & II	138	37.13	38.63
	Modic Absent	85	34.90	35.29
% NRS Change 1 Week	Modic I & II	134	37.78	48.59
	Modic Absent	84	43.93	30.53
% NRS Change 1 Month	Modic I & II	131	26.09	51.17
	Modic Absent	81	36.97	36.89

*N* number of patients, *NRS* numerical rating scale for pain, *SD* standard deviation

When comparing the mean NRS change scores of patients with MC type I versus patient with MC type II no significance difference in percent change in pain severity was found at all the follow-up periods.

## Discussion

Because there has been a growing research interest in the clinical relevance of Modic changes adjacent to degenerated discs as viewed on MRI [2, 3, 16–18], the purpose of this study was to further this research by assessing how a specific patient population, i.e., chronic patients with the presence of MCs either type I only, type II only or with MCs type I and II together, responded to therapeutic imaging-guided lumbar facet joint injections, as compared to patients without MCs. This is the first study trying to make such a comparison. It was hypothesized that because MCs have been linked with LBP in previous studies [2, 3, 5] patients with MC type I changes may have worse outcomes after therapeutic lumbar facet injections as they may have two sources for their pain – facet joints and bone marrow edema.

The results obtained in this study, which had a fairly large sample size, did not find a clinically relevant link between Modic changes, even type I, and the outcomes after therapeutic lumbar facet injections for any of the data collection time points. It was hypothesized that MC type I patients were likely to respond poorly as their MCs could either be the only pain source or an additional pain source along with the facet articulations [2, 3, 5, 17, 19]. Although the mean NRS change score at 1 month for patients without MCs was 14 % higher than the mean change score for patients with MC type I (i.e., absence of MCs means more pain reduction), this did not quite meet the criteria for statistical significance as the *p* value was 0.07. Perhaps a larger sample size would have resulted in this becoming statistically significant, but that would not necessarily mean that it was clinically relevant as the current sample size included 141 patients with MCs. These results are in contrast to the results reported for lumbar disc herniation patients receiving an imaging-guided therapeutic nerve root block where patients with Modic changes (types I and II together) reported significantly worse outcomes compared to lumbar disc herniation patients without Modic changes at the 1 month data collection time point [20]. Both studies used a very similar methodology and identical outcome measures and data collection time points. However, for the lumbar disc herniation patients the MRI findings were linked to their clinical presentation of specific radiculopathy, whereas with lumbar facet injection patients the clinical symptoms are not necessarily as clear and injections are therefore usually done at the most degenerated joints.

Reporting negative results in research studies is as important as reporting positive results. If replicated, negative studies can prevent unnecessary duplication of such studies in the future so that researchers can focus on other issues.

According to Modic et al. [18] the natural course of type I change is replacement with type II over 14 to 36 months. The latter remained stable over 2 to 3 years follow-up evaluation. The exact etiologic mechanism or mechanisms, while unknown, have been thought related to some type of unusual stresses, micro or macro-instability or microtrauma [19]. Recent studies suggest a genetic predisposition in patients showing the presence of MCs at the same level of disc degeneration or disc herniation [21, 22]. Moreover, Kjaer et al. [2] suggested that disc degeneration in the presence of MCs has a specific clinical profile and thus they concluded that a degenerated disc per se is a fairly quiet disorder but it constitutes a true clinical entity when MCs are also present. Unfortunately, they considered type I and type II Modic changes together for the purpose of their study, so no more specific conclusion could be drawn.

It has been found that MC type I are related to non-specific LBP and degenerative disc disease [2, 3, 5, 17, 19] but they have never been directly linked to pain arising from the facet joints. The starting hypothesis that patients with MC type I may have a less favourable outcome from therapeutic imaging-guided facet joint injections, arose from the idea that in those patients part of the LBP may actually arise from the inflammatory changes in the disco-vertebral region of the spine instead of, or in addition to, the lumbar facet articulations. Thus a procedure direct to the zygapophysial joints will not be as effective in decreasing pain. The MCs identified on the MRI scans of these patients were not necessarily at the same level as the facet injections. This is not considered a limitation however as the purpose was to determine whether the presence of MCs anywhere in the lumbar spine may be another source of the patient’s LBP. Additionally, the presence or absence of edema at the injected facet joints themselves was also not investigated as, although facet joint edema has been observed [23] this is not nearly as common as MCs at the discovertebral junctions. The number of patients with findings of facet joint edema in this current study would have been very small. It would therefore require a very large sample size to obtain sufficient patient numbers with lumbar facet joint edema to assess outcomes when considering the frequency of this finding.

### Limitations

Only approximately one third of the patients in this study reported clinically significant improvement (PGIC scores) after the therapeutic facet joint injections. Hence, this reinforces the conflicting literature about non-specific LBP and lumbar facet joint injections. It is important to take into consideration that this current study was based on the hypothesis that the patients’ pain arose from their facet joints, but no test, such as anesthetic blocks of the lumbar medial branches, was performed to really identify the source of pain. The selection of the relevant facet articulations was based on imaging findings of the most severely degenerated facet levels. Whether or not this is correct needs further investigation. However, if the Modic changes were the main pain source, this should not have influenced the results. Usually, just after a lumbar facet joint injection into a painful joint the perceived pain disappears quickly, due to the action of the anesthetic, the real steroid effect is noted after a few days and it can last from 1–2 months to 1–2 years. Interestingly, Manchikanti et al. [24, 25] found an equal effectiveness of local anesthetics with or without steroid, indicating a lack of support for the proposition of inflammation in the pain arising from the lumbar facet joints.

Having a longer follow-up time period was not considered necessary for this current study because if the presence of Modic changes was an additional or significant source of the patients’ low back pain, these Modic positive patients should have reported worse outcomes after the facet joint injections within this 1 month time frame. The anesthetic and corticosteroid medications do not reach the disc and adjacent vertebral bodies when only injected into the facet articulations and thus could not affect the region of the Modic changes. The protocol used for this current study, including the time periods for the data collection, is also very similar to other published studies [20, 26]. If effective, the local anesthetic reduces the pain quickly (i.e., 1 day outcomes) whereas the effects of the corticosteroid injected into the facet articulations, would appear at the 1 week and 1 month data collection time points. If the corticosteroid was going to work, it would have worked by 1 month in the targeted injection site.

Including additional outcome measures such as a larger disability questionnaire would be desirable. However, over-all disability is part of the Patient’s Global Impression of Change’ scale rating and asking the patient to rate their level of function as part of over-all improvement is in the instructions to the patient. This radiology department tested using the Oswestry questionnaire during a pilot study prior to starting the large outcomes database. It was found that this relatively short questionnaire was still too time consuming and disruptive to the flow of this busy department.

Although mistakes could have been made during the interpretation of the MRI studies due to the fact that to define MCs the study is based only on visual impression, the reliability results for detecting and classifying MCs obtained in this study are good, showing a high “substantial” level of inter and intra-examiner reliability, similar to the results obtained from the study of Peterson et al. [27]. Having reached substantial agreement between the two examiners, gives more credibility to the results of the data collected and the data analysis process.

Lastly, the main problems with prospective cohort studies are modifying factors. In this particular study potential modifying factors were likely to be present. These include the fact that information about the chronicity or the reason/cause of patients’ low back pain were not known and are difficult to reliably distract from all patient files. However, as the study took place at a specialized university orthopedic hospital where difficult and chronic cases are referred, it is safe to conclude that the vast majority of these lumbar facet injection patients would be in the chronic category. However, there remained aspects of the patients that could have positively or negatively influenced the outcomes, but that we could not evaluate with the present study. It would have been much better if patients could have been categorized into acute and chronic low back pain and the analyses done separately for each group. Moreover, as already stated above, facet joint syndrome is very difficult to define clinically, therefore for similar future studies it will be important to identify patients with pain really coming from the lumbar facet joints to better evaluate this therapeutic option.

## Conclusions

Although patients without Modic changes showed a tendency toward better outcomes compared to patients with Modic changes, this did not reach statistical significance in a fairly large patient population. Therefore, the present study does not support the hypothesis that LBP patients undergoing imaging-guided lumbar facet injections who also have MC type I have a less favourable outcome from this treatment. Specifically, no statistically and thus clinically significant results, either positive or negative, were found when comparing patients with MCs, either MC type I only, MC type II only or MCs type I and II together, to patients without MCs in terms of improvement and decreased/increased perception of pain. Clinically, this means that the effectiveness of therapeutic lumbar facet joint injections is not altered by the presence or absence of MCs. More studies need to be conducted on the importance of MCs and non-specific LBP with other possible therapeutic options.

## Abbreviations

CI: Confidence interval
EK: Ethics kommission (German)
K: Kappa statistic
LBP: Low back pain
MC: Modic change
MR: Magnetic resonance
MRI: Magnetic resonance imaging
NRS: Numerical rating scale (for pain)
OR: Odds ratio
PGIC: Patients global impression of change
RCT: Randomized clinical trial
SD: Standard deviation
SPSS: Statistical package for the social sciences
